# Unmasking Snake Venom of *Bothrops leucurus*: Purification and Pharmacological and Structural Characterization of New PLA_2_ Bleu TX-III

**DOI:** 10.1155/2013/941467

**Published:** 2013-01-09

**Authors:** Fábio André Marangoni, Luis Alberto Ponce-Soto, Sergio Marangoni, Elen Cristina Teizem Landucci

**Affiliations:** ^1^Department of Pharmacology, Faculty of Medical Sciences, State University of Campinas, 13083-970 Campinas, SP, Brazil; ^2^Department of Biochemistry, Institute of Biology, State University of Campinas, 13083-970 Campinas, SP, Brazil

## Abstract

Bleu TX-III was isolated from *Bothrops leucurus* snake venom on one-step analytical chromatography reverse phase HPLC, was homogeneous on SDS-PAGE, and was confirmed by Q-Tof Ultima API ESI/MS (TOF MS mode) mass spectrometry in 14243.8 Da. Multiple alignments of Bleu TX-III show high degree of homology with basic PLA_2_ myotoxins from other *Bothrops* venoms. Our studies on local and systemic myotoxicity “*in vivo*” reveal that Bleu TX-III is myotoxin with local but not systemic action due to the decrease in the plasmatic CK levels when Bleu TX-III is administrated by intravenous route in mice (dose 1 and 5 **μ**g). And at a dose of 20 **μ**g myotoxin behaves like a local and systemic action. Bleu TX-III induced moderate marked paw edema, evidencing the local increase in vascular permeability. The inflammatory events induced in the mice (I.M.) were investigated. The increase in the levels of IL-1, IL-6, and TNF-**α** was observed in the plasma. It is concluded that Bleu TX-III induces inflammatory events in this model. The enzymatic phospholipid hydrolysis may be relevant to these phenomena. *Bothrops leucurus* venom is still not extensively explored, and the knowledge of its toxins separately through the study of structure/function will contribute for a better understanding of its action mechanism.

## 1. Introduction

Snake venom contains a mixture of powerful proteins and peptides that have evolved to be targeted to receptors, ion channels, or enzymes [1], in addition to some carbohydrates, nucleosides, lipids, and metal ions, whose functions are not all known [2, 3]. They interact with a wide variety of mammalian proteins and can disrupt the central and peripheral nervous systems, the blood coagulation cascade, the cardiovascular and neuromuscular systems, and homeostasis in general. These venom proteins act with great precision different toxins recognize different subtypes of certain receptors with only subtle differences and are very biologically active.

Phospholipases A_2_ (PLA_2_, EC 3.1.1.4) are generally Ca^2+^-dependent enzymes that catalyze the hydrolysis of the sn-2 fatty acyl bond of glycerophospholipids. Secreted PLA_2_s are small proteins (14–18 kDa) usually containing 5–8 disulfide bonds and possessing a His/Asp dyad required for catalysis. Snake venom PLA_2_s are classified into groups I or II, based on their sequence and mode of disulphide pairings. Group I PLA_2_s are found in the venoms of Elapidae snakes, whereas group II PLA_2_s are present in the venoms of Viperidae snakes [4]. The group II is further divided into two main subgroups: Asp49 and Lys49 (PLA_2_ homologues) variants. In the latter, the aspartic acid residue at position 49, critically involved in calcium binding and essential for catalytic activity, is replaced by lysine. Due to this and other critical substitutions, the Lys49 PLA_2_s cannot bind calcium efficiently and are considered to be enzymatically inactive [5, 6]. Although catalytic activity has been shown to play a role in the toxic actions of some venom PLA_2_s, it is not essential in the case of Lys49 PLA_2_s, which use nonenzymatic mechanisms to alter membrane homeostasis [6].

Several PLA_2_s have been identified from *Bothrops leucurus *venom including one acidic [7], one basic phospholipase A_2_ and phospholipase A_2_ homologous K49 [8]. This diversity of PLA_2_, found in the venom of *Bothrops leucurus*, agrees with studies that show marked ontogenetic and individual venom variations [1].

In the present work, a new basic PLA_2_ (Bleu TX-III) from the venom of *Bothrops leucurus* has been isolated and characterized, in order to obtain insights into its possible biological roles and its relevance to the pathophysiology of envenomings by this species in the north-eastern Brazil.

## 2. Material and Methods

### 2.1. Reverse Phase HPLC

The PLA_2_ (Bleu TX-III) from *Bothrops leucurus* snake venom was purified by reverse phase HPLC according to method described by Ponce-Soto et al. [9]. Briefly, 5 mg of whole venom was dissolved in 100 *μ*L of buffer A (0.1% TFA) and 150 *μ*L NH_4_HCO_3_ 50 mM and centrifuged at 4500 g, and the supernatant was then applied on the analytical reverse phase HPLC *μ*-Bondapack C-18, previously equilibrated with buffer A for 15 min. The elution of the protein was then conducted using a linear gradient of buffer B (66.5% Acetronitrile in buffer A), and the chromatographic run was monitored at 280 nm of absorbance. After elution the fraction was lyophilized and stored at 40°C.

### 2.2. Electrophoresis

Tricine SDS-PAGE in a discontinuous gel and buffer system was used to estimate the molecular mass of the proteins, under reducing and nonreducing conditions [10].

### 2.3. PLA_2_ Activity

PLA_2_ activity was measured using the assay described [11] modified for 96-well plates [12]. The standard assay mixture contained 200 *μ*L of buffer (10 mM Tris-HCl, 10 mM CaCl_2_, and 100 mM NaCl, pH 8.0), 20 *μ*L of substrate (3 mM), 20 *μ*L of water and 20 *μ*L of PLA_2_ (1 mg/mL) in a final volume of 260 *μ*L. After adding PLA_2_ (Bleu TX-III) from *Bothrops leucurus* (20 *μ*g), the mixture was incubated for up to 40 min at 37°C, with the reading of absorbance at intervals of 10 min. The enzyme activity, expressed as the initial velocity of the reaction (*V*_*o*_), was calculated based on the increase of absorbance after 20 min. All assays were done in triplicate, and the absorbances at 425 nm were measured with a VersaMax 190 multiwell plate reader (Molecular Devices, Sunnyvale, CA, USA).

### 2.4. Amino Acid Analysis

Amino acid analysis was performed on a Pico-Tag Analyzer (Waters Systems) as described [13]. The PLA_2_ (Bleu TX-III) from *Bothrops leucurus*, sample (30 *μ*g), was hydrolyzed at 105°C for 24 h, in 6 M HCl (Pierce sequencing grade) containing 1% phenol (w/v). The hydrolyzates were reacted with 20 *μ*L of derivatization solution (ethanol : triethylamine : water : phenylisothiocyanate, 7 : 1 : 1 : 1, v/v) for 1 h at room temperature, after which the PTC-amino acids were identified and quantified by HPLC, by comparing their retention times and peak areas with those from a standard amino acid mixture.

### 2.5. Mass Spectrometry

An aliquot (4.5 *μ*L) of the modified proteins was inject in C18 (100 *μ*m × 100 mm) RP-UPLC (nanoAcquity UPLC, Waters) coupled with nanoelectrospray tandem mass spectrometry on a Q-Tof Ultima API mass spectrometer (MicroMass/Waters) at a flow rate of 600 nl/min. The gradient was 0–50% acetonitrile in 0.1% formic acid over 45 min. The instrument was operated in MS continuum mode, and the data acquisition was from *m/z* 100–3.000 at a scan rate of 1 s and an interscan delay of 0.1 s. The spectra were accumulated over about 300 scans and the multiple charged data by the mass spectrometer on the *m/z* scale were converted to the mass (molecular weight) scale using Maximum Entropy-based software supplied with Masslynx 4.1 software package. The processing parameters were output mass range 6.000–20.000 Da at a “resolution” of 0.1 Da/channel; the simulated isotope pattern model was used with the spectrum blur width parameter set to 0.2 Da, the minimum intensity ratios between successive peaks were 20% (left and right). The deconvoluted spectrum was then smoothed (2 × 3 channels, Savitzky Golay smooth) and the mass centroid values obtained using 80% of the peak top and a minimum peak width at half height of 4 channels.

### 2.6. Myotoxic Activity

Groups of four Swiss mice (18–20 g) received an intramuscular (i.m.) or an intravenous (i.v.) injection of variable amounts of PLA_2_ (Bleu TX-III) from *Bothrops leucurus* in 100 *μ*L of PBS, in the gastrocnemius. A control group received 100 *μ*L of PBS. At different intervals of time (2, 4, 6, 9, and 24 h) blood was collected from the tail into heparinized capillary tubes, and the plasma creatine kinase (CK; EC 2.7.3.2) activity was determined by a kinetic assay (Sigma 47-UV). Activity was expressed in U/L, one unit defined as the phosphorylation of 1 *μ*mol of creatine/min at 25°C.

### 2.7. Edema-Forming Activity

The ability of PLA_2_ (Bleu TX-III) from *Bothrops leucurus* to induce edema was studied in groups of five Swiss mice (18–20 g). 50 *μ*L of phosphate-buffered saline (PBS; 0.12 M NaCl, 0.04 M sodium phosphate, pH 7.2) with venom (10 *μ*g/paw) was injected in the subplantar region of the right footpad. The control group received an equal volume of PBS alone. The swelling of the paw was measured at 0.5, 1, 3, 6, 9, and 24 h after administration. Edema was expressed as the percentage increased in the volume of the treated group to that of the control group at each time interval.

### 2.8. Cytokines

The levels of cytokines IL-6 and IL-1 in the serum from BALB/c mice were assayed by a two-site sandwich enzyme-like immunosorbent assay (ELISA) as described [14]. In brief, ELISA plates were coated with 100 *μ*L (1 *μ*g/mL) of the monoclonal antibodies anti-IL-6 and anti-IL-1. In 0.1 M sodium carbonate buffer (pH 8.2) and incubated for 6 hours at room temperature. The wells were then washed with 0.1% phosphate-buffered saline (PBS/Tween-20) and blocked with 100 *μ*L of 10% fetal calf serum (FCS) in PBS for 2 hours at room temperature. After washing, duplicate sera samples of 50 *μ*L were added to each well. After 18 hours of incubation at 4°C, the wells were washed and incubated with 100 *μ*L (2 *μ*g/mL) of the biotinylated monoclonal antibodies anti-IL-6 and anti-IL-1 as second antibodies for 45 minutes at room temperature. After a final wash, the reaction was developed by the addition of orthophenyldiamine (OPD) to each well. Optical densities were measured at 405 nm in a microplate reader. The cytokine content of each sample was read from a standard curve established with the appropriate recombinant cytokines (expressed in picograms per millilitre). The minimum levels of each cytokine detectable in the conditions of the assays were 10 pg/mL for IL-6 and IL-1.

To measure the cytotoxicity of TNF-*α* present in the serum from BALB/c mice, a standard assay with L-929 cells, a fibroblast continuous cell line was used as described previously [15]. The percentage cytotoxicity was calculated as follows:

(1) $$\begin{array}{c} \left(A_{\text{control}}-\frac{A_{\text{sample}}}{A_{\text{control}}}\right)\times 100. \end{array}$$

Titres were calculated as the reciprocal of the dilution of the sample in which 50% of the cells in the monolayer were lysed. TNF-*α* activity is expressed as units/mL, estimated from the ratio of a 50% cytotoxic dose of the test to that of the standard mouse recombinant TNF-*α*.

### 2.9. Statistical Analyses

Results were reported as mean ± SEM. The significance of differences among means was assessed by analysis of variance followed by Dunnett's test when several experimental groups were compared with the control group. Differences were considered statistically significant if *P* < 0.05.

## 3. Results

**Figure 1:**
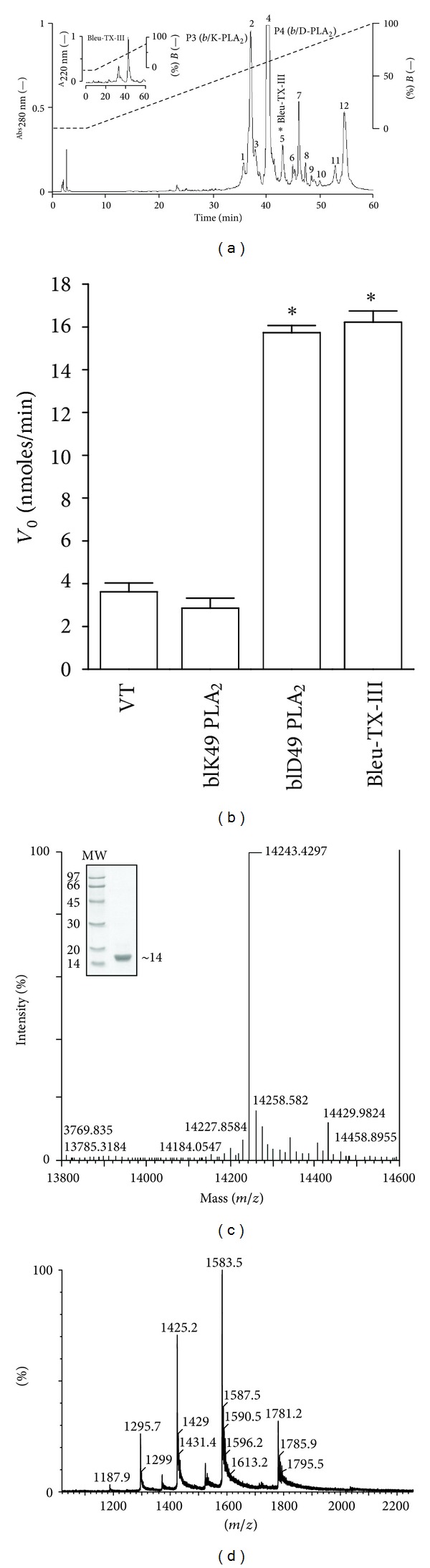
(a) Elution profile of *Bothrops leucurus* venom by RP-HPLC on a *μ*-Bondapack C18 column. *Fraction 5 (PLA_2_ Bleu TX-III) contained PLA_2_ and myotoxic activity. Insert: rechromatography on RP-HPLC of Bleu TX-III. (b) PLA2 activity of *Bothrops leucurus* venom, *b*/K49, *b*/D49 [8], and PLA_2_ Bleu TX-III. The results of all experiments are the mean ± SEM of three determinations (*P* < 0.05). (c) Mass determination of the native Bleu TX-III by Q-Tof Ultima API ESI/MS (TOF MS mode) mass spectrometry. Insert electrophoretic profile in Tricine SDS-PAGE. (d) Raw electrospray-positive mass spectrum, showing multicharged ions distributions of native Bleu TX-III.

The elution profile of *Bothrops leucurus* venom following RP-HPLC performed on a C18 column showed thirteen fractions (1–12) (Figure 1(a)). The five eluted peaks were screened for PLA_2_ activity. Only the fraction labeled in Figure 1(a) presented PLA_2_ activity, which was eluted with 59% of buffer B. This peak was further purified by the same chromatography system used in the first fractionation step (RP-HPLC). The result of the repurification showed the presence of only one peak, named Bleu TX-III (Figure 1(a) inserted).

The Q-Tof Ultima API ESI/MS (TOF MS mode) mass spectrometry analysis confirmed the homogeneity of the fraction Bleu TX-III and determined the exact molecular mass of 14243, 4297 Da (Figure 1(b)). This value of molecular mass was used in calculating the molar concentrations of toxin used in the experiments described below.

The amino acid composition determined was D/11, T/9, S/7, E/9, P/4, G/11, A/5, C/14, V/4, M/3, I/5, L/7, Y/11, F/5, K/7, H/3 e R/10, W/Not determined.

The PLA_2_ activity was examined in the *Bothrops leucurus* venom and in Bleu TX-III using the synthetic substrate 4-nitro-3 (octanoyloxy) benzoic. The PLA_2_ activity was higher in Bleu TX-III (16,22 ± 0,5268 nmols/mim/mg) and P4 (*b*/D-PLA_2_) (15,728 ± 0,3354 nmols/mim/mg), when compared with fraction 2 (P3 *b*/K-PLA_2_) (2,856 ± 0,464 nmols/mim/mg), and the whole venom (3,617 ± 0,4144 nmols/min/mg) (Figure 1(c)).

The alkylated protein Bleu TX-III was digested separately with trypsin, and the resulting tryptic peptides were fractionated by RP-HPLC. Each peak numbered in the chromatogram (data not shown) was manually collected and lyophilized, and sequencing of the peptide was done by ESI mass spectrometry. Isoleucine and leucine residues were not discriminated in any of the sequences reported since they were indistinguishable in the low-energy CID spectra. Due to the external calibration applied to all spectra, it was also not possible to resolve the 0.036 Da difference between the glutamine and lysine residues, except for the lysine that was deduced based on the cleavage and missed cleavage of the enzyme.

The deduced sequence and measured masses of alkylated peptides of Bleu TX-III are summarized in Table 1; on the basis of sequence determination, 9 peptides were finding in the protein. The sequence of each peptide was then submitted separately to the NCBI database using the protein search program BLAST-p. Using the position matches of the sequenced peptides with homologous proteins present in the database, it was possible to deduce their original position on the unknown protein Bleu TX-III.

The sequence of those proteins returns high homology with the sequence of a phospholipase A_2_ present in the venom of *Crotalus scutulatus scutulatus* (Mojave rattlesnake) (PA2B_CROSS Accession Number P62023; [16]. The partial Bleu TX-III sequence obtained was then resubmitted to BLAST-p, with the search restricted to Crotalinae snakes.  Figure 3 shows the result of BLAST alignment between Bleu TX-III with the phospholipase A_2_ from human pancreatic (0910150a) [17] and other PLA_2_ coming from the venom of snakes of the family Viperidae. P62023 Mtx-b PLA_2_ of *Crotalus scutulatus scutulatus* [16]; P0C942_1a LmTX-I de *Lachesis muta muta* [5] and P0C8M1 BmTX-I of *Bothrops moojeni* [18].

**Figure 2:**
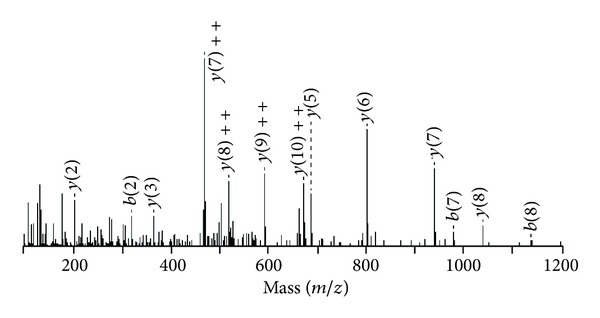
MS/MS spectrum of the doubly-charged tryptic ion of *m/z* 1504. Ion of the major sequence-specific *y*-ion serie and of aminor series of the complementing *b*-ions CCFVHDCCYGK, from which the sequence of Bleu TX-III tag was deduced.

The sequence coverage was high for Bleu TX-III; the protein shared the conserved sequence domains common to this group of proteins, including the 14 cysteines, the calcium-binding site located on Gly30, Gly32, Tyr28, and Asp49, together with the amino acid of active site His48 (SwissProt database http://br.expasy.org/). The tandem mass spectra shown in Figure 2, relative to the peptide eluted in fraction 3 of both digest, having the sequence KCCFVHDCCYG, allow to classify both enzymes as PLA_2_.

*In vivo*, the Bleu TX-III induced a visible local myotoxic when injected by the i.m. route (Figure 4(a)), but with a regular increase in plasma levels of CK occurred after IV injection in single dose of 20 *μ*g. Time-course analysis showed a maximum increase in plasma CK 3 hours after i.m. injection, returning to normal by 24 h (Figure 4(b)).

Bleu TX-III also induced moderate footpad edema, with a MED of 5 ± 2 *μ*g, evidencing the local increase in vascular permeability. Edema reached its highest point after 3 h, rapidly returning to basal levels thereafter (Figure 4(c)).

To further analyse and compare the mechanisms of the inflammatory events induced by PLA_2_ Bleu TX-III, the concentrations of IL-1, IL-6, and TNF-*α* in the plasma were measured. Bleu TX-III caused a marked increase in levels of both IL-1 and IL-6, 1, 3, and 6 hours, peaking at 6 hours for IL-1 and 3 hours for IL-6, respectively, (Figures 4(d) and 4(e)). Bleu TX-III caused a significant increase in the TNF-*α* concentrations only at 1 h (Figure 4(f)).

## 4. Discussion

PLA_2_s are among the most abundant components of snake venoms, showing a wide array of activities in spite of a conserved overall structure [19]. Understanding the structural basis for their diverse toxic activities, including myotoxicity and inflammatory, is still a challenging task. In this work, a new toxin was isolated, and structurally and functionally characterized, from *Bothrops leucurus* venom, showing that it belongs to the family of PLA_2_.

The purification procedure for basic PLA_2_s developed [9, 20, 21] is shown to be also efficient for the obtainment of the PLA_2_ Bleu TX-III myotoxin from *Bothrops leucurus* snake venom. Fractionation protocol of this crude venom using a single pass chromatographic in a column *μ*-Bondapack C-18 coupled to a system of reverse phase HPLC gave rise to 12 fractions at 280 nm, the two last being the basic myotoxins, named Bleu TX-III PLA_2_ (5). This rapid procedure showed high yield, producing 5–10 mg of the proteins with high purity levels (Figure 1(a)) and rechromatography of the major peak by RP-HPLC, what has yielded one main peak (Figure 1(a) inserted). The use of NH_4_HCO_3_ and acetonitrile (RP-HPLC) as the buffer system is advantageous since these solvents are easily eliminated by lyophilization, thereby eliminating the need for desalting as in the case of ammonium bicarbonate.

The sequences of several tryptic peptides of peaks 2 and 4 (date not shown) were the same as described for P3 (*bl*/K-PLA_2_) and P4 (*bl*/D49-PLA_2_) [8] (Figure 1(a)). Bleu TX-III was isolated to homogeneity by one chromatographic step. SDS-PAGE under nonreducing conditions showed that it occurs as a monomer, in the range of ~14 kDa after reduction (Figure 1(b) inserted). A subunit molecular mass of 14243.4297 Da was determined by ESI/MS mass spectrometry. The amino acid composition of the toxin revealed a high content of basic and hydrophobic residues, with 14 half-Cys, in agreement with the reported compositions and primary structures of myotoxic PLA_2_s isolated from *Bothrops* venoms [9, 21–23].

This basic PLA_2_ showed enzymatic activity on monodisperse substrate, with a strict requirement of Ca^2+^, and maximum activity at pH 8.0 and 40°C. These characteristics are common to other bothropic and crotalic PLA_2_s [21, 24, 25]. The PLA_2_ activity is suggested to be higher in Bleu TX-III (16.22 ± 0.5268 nmols/mim) and *bl*/D-49 (4) (15.728 ± 0,3354 nmoles/min) when compared with the whole venom (3.617 ± 0.4144 nmoles/min) and *bl*/K-49 (2.856 ± 0.464 nmoles/min) (Figure 1(c)). The Ca^2+^ ion dependent on the enzymatic activity of Bleu TX-III revealed that the Ca^2+^ ion is an obligatory cofactor for its enzymatic activity. This can be explained by different coordination geometries assumed by the tetrahedral intermediate due to the presence of the Ca^2+^ ion which determine the electrophilic behavior of the catalytic site, as well as stabilizes the otherwise flexible Ca^2+^-binding loop and appears to optimize the interaction enzyme substrate [26].

**Figure 3:**
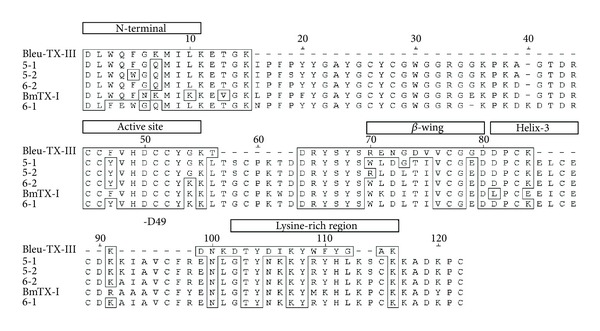
Alignment of the deduced amino acid sequence of the Bleu TX-III PLA_2_ with D49-PLA_2_. 5_I and 5_II (BmjeTX-I and BmjeTX-II) from *Bothrops marajoensis* [28], 6_1 and 6_2 from *Bothrops jararacussu* [20] and BmTX-I from *Bothrops moojeni* [18].

Comparison of the N-terminal sequence of Bleu TX-III showed similarity with other myotoxic PLA_2_ from *Bothrops* genus (Figure 3). 

To Bleu TX-III, no substitution was found in the conserved regions of the catalytic activity, as the channel hydrophobic N-terminal region (1 to 10) is important as a part of the interfacial bonding surface, so as active site (44 to 57) as suggested [26–28]. Considering the 7-peptides sequenced (this work), Bleu TX-III, showed high-level homology with many PLA_2_, from different snakes species. The highly conserved sequences XCGXGG and DXCCXXHD responsible for the Ca^2+^-binding loop and the catalytic site of PLA_2_ [29], respectively, are present in the sequence of PLA_2_ Bleu TX-III.

The P3 (*bl*/K-PLA_2_), P4 (*bl*/D-PLA_2_), and fraction 5 (Bleu TX-III) enzymes seemed to be completely separated by reverse phase chromatography, and when some triptic peptides were sequenced by ESI/MS, of three PLA_2_, venom showed that *Bothrops leucrurus* have several homologous PLA_2_ and PLA_2_.

As a result of complications, mainly local edema and necrosis, usually occurred following ophidian accidents with *Bothrops* snakes [30, 31], and studies involving PLA_2_s became very important, since they are the main venom components responsible for necrosis and inflammatory response [32].

Histological and ultrastructural studies of the effect of venom PLA_2_s on skeletal muscle reveal a common series of pathological alterations which include (1) plasma membrane disruption, (2) formation of “delta-lesions,” wedge-shaped areas of degeneration located at the periphery of muscle fibers, (3) hypercontraction of myofilaments, (4) mitochondrial swelling, together with the formation of flocculent densities and rupture of mitochondrial membranes, (5) disruption of intracellular membrane systems, that is, sarcoplasmic reticulum and T tubules, and (6) pycnosis of nuclei [32–35].

**Figure 4:**
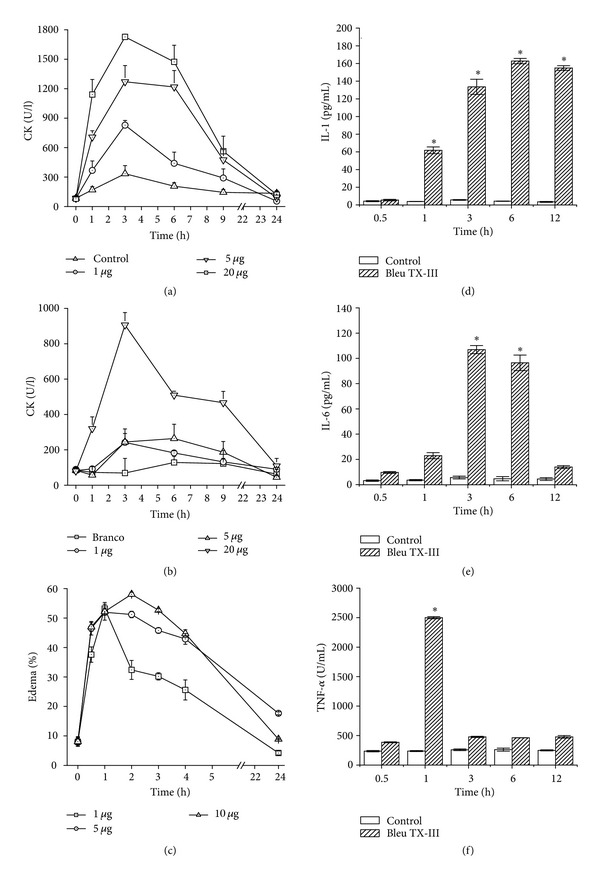
(a) and (b) Time-course of the increments in plasma CK activity after intramuscular injection and intravenous of Bleu TX-III PLA_2_ (1, 5, and 20 *μ*g). Controls were injected with 100 *μ*L of PBS. At different times, blood was collected, and serum levels were measured. Values are means ± SEM of five mice at each time point. (c) Edema-forming activity of Bleu TX-III PLA_2_ (1, 5 and 20 *μ*g). In mice. Induction of edema by Bleu TX-III PLA_2_, injected in the footpad of mice. At various time intervals the increase in footpad volume, as compared to controls, was expressed as percent edema. Each point represents the mean ± SEM of five animals. (d), (e), and (f) Cytokines levels in mice. The production of IL-1, IL-6, and TNF-*α* was assayed in plasma of Bleu TX-III PLA_2_. The experiments were performed in triplicate and analyzed statistically by ANOVA or Kruskal-Wallis tests and confirmed by Tukey and Tukey-type tests. Each point represents ±SEM of seven mice *P* < 0.05 between the experimental groups and control group.

Our studies on local and systemic myotoxicity *in vivo* show that PLA_2_ Bleu TX-III is not systemic myotoxins in the dose of 5 *μ*g and 20 *μ*g systemic slightly as local action due to decreased plasma levels of CK (Figures 4(a) and 4(b)). This reinforces the hypothesis of differential action of local and systemic myotoxicity proposed [32] and also the specificity and specificity proposed [21, 23, 28].

The snake venom *Bothrops* induced local edema in humans and experimental animals [36]. Besides Bleu TX-III, a number of snake venom PLA_2_s, others also induce edema of 30 minutes after injection (Figure 3(c)) [4, 37, 38]. Studies have been conducted trying to understand the mechanisms involved in the inflammatory response induced by the myotoxic PLA_2_ from several snake venoms [30]. Studies have been directed trying to understand the mechanisms involved in the inflammatory response induced by myotoxic PLA_2_ from several snake venoms [39–41]. However, the relationship between enzymatic activity and edema is contradictory [42]. It is assumed that myotoxic and edematogenic activities can be induced by different structural domains in these PLA_2_, or that a partial overlapping of these domains occur [43].

Cytokines, such as IL-1, IL-6, and TNF-*α*, are also relevant mediators for leukocyte migration and participate in several inflammatory conditions. Our results showed that PLA_2_ Bleu TX-III induced a stronger effect on the expression of adhesion molecules by endothelial cells and stimulates the release of both IL-1, IL-6, and TNF-*α* [44]. Thus, our results suggest that IL-1 may contribute for the leukocyte (Figures 3(d), 3(e), and 3(f)).

In conclusion, Bleu TX-III induces a marked inflammatory reaction in the mouse serum. Since basic myotoxic PLA_2_s are abundant in snake venoms, these toxins must play a relevant role in the proinflammatory activity that characterizes this venom. The fact that Bleu TX-III elicited a stronger reaction inflammatory argues in favor of a role of enzymatic phospholipid hydrolysis in this phenomenon, either through the direct release of arachidonic acid from plasma membranes or through the activation of intracellular processes in target cells.

## Figures and Tables

**Table 1:** Sequence obtained by ESI-MS/MS based on the alkylated tryptic peptides derived. The peptide were separated and sequenced by mass spectrometry.

Residue Number	Mass (Da) expected	Amino acid sequence	Mass (Da) calculated
1–11	1377.6510	DLWQFGKMILK	1377.7479
8–15	918.4353	MILKETGK	918.5208
43–53	1504.4404	CCFVHDCCYGK	1504.5356
61–68	1046.4846	TDRYSYSR	1046.4781
69–82	1520.6066	ENGDVVCGGDDPCK	1520.5872
98–106	1110.4915	DNKDTYDIK	1110.5193
107–113	933.4279	YWFYGAK	933.4385

## References

[1] Calvete JJ (2009). Venomics: digging into the evolution of venomous systems and learning to twist nature to fight pathology. *Journal of Proteomics*.

[2] Fox JW, Serrano SMT (2008). Exploring snake venom proteomes: multifaceted analyses for complex toxin mixtures. *Proteomics*.

[3] Georgieva D, Arni RK, Betzel C (2008). Proteome analysis of snake venom toxins: pharmacological insights. *Expert Review of Proteomics*.

[4] Chaves F, León G, Alvarado VH, Gutiérrez JM (1998). Pharmacological modulation of edema induced by Lys-49 and Asp-49 myotoxic phospholipases A_2_ isolated from the venom of the snake *Bothrops asper* (terciopelo). *Toxicon*.

[5] Damico DCS, Lilla S, De Nucci G (2005). Biochemical and enzymatic characterization of two basic Asp49 phospholipase A_2_ isoforms from *Lachesis muta muta* (Surucucu) venom. *Biochimica et Biophysica Acta*.

[6] Doley R, Kini RM (2009). Protein complexes in snake venom. *Cellular and Molecular Life Sciences*.

[7] Nunes DCO, Rodrigues RS, Lucena MN (2011). Isolation and functional characterization of proinflammatory acidic phospholipase A_2_ from *Bothrops leucurus* snake venom. *Comparative Biochemistry and Physiology C*.

[8] Higuchi DA, Barbosa CMV, Bincoletto C (2007). Purification and partial characterization of two phospholipases A_2_ from *Bothrops leucurus* (white-tailed-jararaca) snake venom. *Biochimie*.

[9] Ponce-Soto LA, Baldasso PA, Romero-Vargas FF, Winck FV, Novello JC, Marangoni S (2007). Biochemical, pharmacological and structural characterization of two PLA_2_ isoforms Cdr-12 and Cdr-13 from *Crotalus durissus ruruima* snake venom. *Protein Journal*.

[10] Schagger H, Von Jagow G (1987). Tricine-sodium dodecyl sulfate-polyacrylamide gel electrophoresis for the separation of proteins in the range from 1 to 100 kDa. *Analytical Biochemistry*.

[11] Holzer M, Mackessy SP (1996). An aqueous endpoint assay of snake venom phospholipase A_2_. *Toxicon*.

[12] Ponce-Soto LA, Toyama MH, Hyslop S, Novello JC, Marangoni S (2002). Isolation and preliminary enzymatic characterization of a novel PLA_2_ from *Crotalus durissus collilineatus* venom. *Journal of Protein Chemistry*.

[13] Hendrickson RL, Meredith SC (1984). Amino acid analysis by reverse-phase high-performance liquid chromatography: precolumn derivatization with phenylisothiocyanate. *Analytical Biochemistry*.

[14] Schumaker JH, OGarra A, Schrader B (1988). The characterization of four monoclonal antibodies specific for mouse IL-5 and development of mouse and human IL-5 ELISA. *The Journal of Immunology*.

[15] Ruff MR, Gifford GE (1980). Purification and physico-chemical characterization of rabbit tumor necrosis factor. *Journal of Immunology*.

[16] John TR, Smith LA, Kaiser II (1994). Genomic sequences encoding the acidic and basic subunits of Mojave toxin: unusually high sequence identity of non-coding regions. *Gene*.

[17] Verheij HM, Westerman J, Sternby B, de Haas GH (1983). The complete primary structure of phospholipase A_2_ from human pancreas. *Biochimica et Biophysica Acta*.

[18] Calgarotto AK, Damico DCS, Ponce-Soto LA (2008). Biological and biochemical characterization of new basic phospholipase A_2_ BmTX-I isolated from *Bothrops moojeni* snake venom. *Toxicon*.

[19] Valentin E, Lambeau G (2000). What can venom phospholipases A_2_ tell us about the functional diversity of mammalian secreted phospholipases A_2_?. *Biochimie*.

[20] Ponce-Soto LA, Bonfim VL, Rodrigues-Simioni L, Novello JC, Marangoni S (2006). Determination of primary structure of two isoforms 6-1 and 6-2 PLA 2 D49 from *Bothrops jararacussu* snake venom and neurotoxic characterization using in vitro neuromuscular preparation. *Protein Journal*.

[21] Ponce-Soto LA, Barros JC, Marangoni S (2009). Neuromuscular activity of BaTX, a presynaptic basic PLA_2_ isolated from *Bothrops alternatus* snake venom. *Comparative Biochemistry and Physiology C*.

[22] Gutiérrez J, Lomonte B (1995). Phospholipase A_2_ myotoxins from Bothrops snake venoms. *Toxicon*.

[23] Kini RM (2003). Excitement ahead: structure, function and mechanism of snake venom phospholipase A_2_ enzymes. *Toxicon*.

[24] Huancahuire-Vega S, Ponce-Soto LA, Martins-de-Souza D, Marangoni S (2009). Structural and functional characterization of brazilitoxins II and III (BbTX-II and -III), two myotoxins from the venom of *Bothrops brazili* snake. *Toxicon*.

[25] Pereañez JA, Núñez V, Huancahuire-Vega S, Marangoni S, Ponce-Soto LA (2009). Biochemical and biological characterization of a PLA_2_ from crotoxin complex of *Crotalus durissus cumanensis*. *Toxicon*.

[26] Scott DL, White SP, Otwinowski Z, Yuan W, Gelb MH, Sigler PB (1990). Interfacial catalysis: the mechanism of phospholipase A_2_. *Science*.

[27] Arni RK, Ward RJ (1996). Phospholipase A_2_—a structural review. *Toxicon*.

[28] Ponce-Soto LA, Martins-De-souza D, Marangoni S (2010). Neurotoxic, myotoxic and cytolytic activities of the new basic PLA_2_ isoforms BmjeTX-I and BmjeTX-II isolated from the *Bothrops marajoensis* (marajó lancehead) snake venom. *Protein Journal*.

[29] Renetseder R, Brunie S, Dijkstra BW, Drenth J, Sigler PB (1985). A comparison of the crystal structure of phospholipase A_2_ from bovine pancreas and *Crotalus atrox* venom. *Journal of Biological Chemistry*.

[30] Gutiérrez JM (2002). Understanding snake venoms: 50 years of research in Latin America. *Revista De Biologia Tropical*.

[31] Kamiguti AS, Cardoso JLC, Theakston RDG (1991). Coagulopathy and haemorrhage in human victims of *Bothrops jararaca* envenoming in Brazil. *Toxicon*.

[32] Gutiérrez JM, Ownby CL (2003). Skeletal muscle degeneration induced by venom phospholipases A 2: insights into the mechanisms of local and systemic myotoxicity. *Toxicon*.

[33] Gutierrez JM, Ownby CL, Odell GV (1984). Skeletal muscle regeneration after myonecrosis induced by crude venom and a myotoxin from the snake *Bothrops asper* (Fer-de-Lance). *Toxicon*.

[34] Harris JB, Cullen MJ (1990). Muscle necrosis caused by snake venoms and toxins. *Electron Microscopy Reviews*.

[35] Mebs D, Ownby CL (1990). Myotoxic components of snake venoms: their biochemical and biological activities. *Pharmacology and Therapeutics*.

[36] Gutiérrez JM, Chaves F, Cerdas L (1986). Inflammatory infiltrate in skeletal muscle injected with *Bothrops asper* venom. *Revista de Biologia Tropical*.

[37] Ketelhut DFJ, Homem De Mello M, Veronese ELG (2003). Isolation, characterization and biological activity of acidic phospholipase A_2_ isoforms from *Bothrops jararacussu* snake venom. *Biochimie*.

[38] Rodrigues RS, Izidoro LFM, Teixeira SS (2007). Isolation and functional characterization of a new myotoxic acidic phospholipase A_2_ from *Bothrops pauloensi*s snake venom. *Toxicon*.

[39] Landucci ECT, Castro RC, Pereira MF (1998). Mast cell degranulation induced by two phospholipase A_2_ homologues: dissociation between enzymatic and biological activities. *European Journal of Pharmacology*.

[40] Landucci ECT, De Castro RC, Toyama M (2000). Inflammatory oedema induced by the Lys-49 phospholipase A_2_ homologue piratoxin-I in the rat and rabbit. Effect of polyanions and *p*-bromophenacyl bromide. *Biochemical Pharmacology*.

[41] Lomonte B, Tarkowski A, Hanson LA (1993). Host response to *Bothrops asper* snake venom. Analysis of edema formation, inflammatory cells, and cytokine release in a mouse model. *Inflammation*.

[42] Vishwanath BS, Kini RM, Gowda TV (1987). Characterization of three edema-inducing phospholipase A_2_ enzymes from habu (*Trimeresurus flavoviridis*) venom and their interaction with the alkaloid aristolochic acid. *Toxicon*.

[43] Zuliani JP, Fernandes CM, Zamuner SR, Gutiérrez JM, Teixeira CFP (2005). Inflammatory events induced by Lys-49 and Asp-49 phospholipases A_2_ isolated from *Bothrops asper* snake venom: role of catalytic activity. *Toxicon*.

[44] Stylianou E, Saklatvala J (1998). Interleukin-1. *International Journal of Biochemistry and Cell Biology*.

